# Profiling the effect of low frequency mechanical vibration on the metabolic and oxidative stress responses of A431 carcinoma

**DOI:** 10.1002/2211-5463.70055

**Published:** 2025-05-16

**Authors:** Wresti L. Anggayasti, Chikahiro Imashiro, Takashi Morikura, Shogo Miyata, Akira Funahashi, Kenjiro Takemura

**Affiliations:** ^1^ Graduate School of Brawijaya University Malang Indonesia; ^2^ Department of Precision Engineering The University of Tokyo Tokyo Japan; ^3^ Department of Biosciences and Informatics Keio University Yokohama Kanagawa Japan; ^4^ Department of Mechanical Engineering Keio University Yokohama Kanagawa Japan

**Keywords:** A431 carcinoma, mechanical cancer therapy, metabolic and oxidative stress, ROS

## Abstract

Mechanomedicine represents a potential biocompatible method in cancer therapy. In particular, the use of low‐frequency mechanical vibration previously proved to trigger apoptosis of the human epidermoid carcinoma A431 cell line. In this study, we further characterized the metabolic and oxidative stress responses triggered by 1 h of 20 Hz mechanical vibration stimulus to A431 prior to cell death. Our results indicate that cell death may be related to the decrease of glucose consumption rate and the higher expression of reactive oxygen species right after mechanical stimulation (0 h). The overexpression of *HMGB1* and *HSP70* coding genes signified the increase of A431 cell stress. However, *HMGB1* and *HSP70* expression decreased at 24 h after mechanical vibration, along with the progression of cell death. We also observed cell morphology changes on A431 cells following vibration which might be indicative of A431 death by apoptosis. The emergence of these stress responses suggests that several pathways are connected to promote cancer cell death. The discovery of A431 cellular stress symptoms which lead to apoptotic death may clarify the usefulness of mechanical vibration in cancer treatment as a novel application of biomechanical manipulation.

## Graphical abstract

20 Hz mechanical vibration induced A431 cancer cells' apoptosis without such effect on other healthy cell lines of L929 and C2C12. Lowered glucose consumption is observed specifically in A431. The expressions of ROS, HMGB1, and HSP1 levels initially increase and subsequently decrease in the cancer cell line, as opposed to L929 and C2C12, which displayed no particular stress reactions.

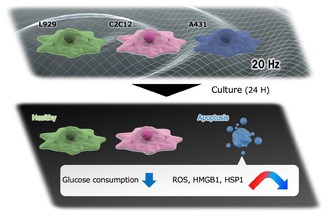

**Abbreviations**
ATP: adenosine triphosphate
DAMP: damage‐associated molecular pattern
DMEM: Dulbecco's modified Eagle's medium
FBS: fetal bovine serum
GAPDH: glyceraldehyde‐3‐phosphate dehydrogenase
GLUT1: glucose transporter 1
HaCat: human keratinocytes
HMGB1: high mobility group box 1
HSP70: Heat Shock Protein 70
LINC: linker of nucleoskeleton and cytoskeleton
MEM: minimal essential medium
mRNA: messenger ribonucleic acid
ROS: reactive oxygen species
RT‐qPCR: quantitative reverse transcription polymerase chain reaction

Various conditions of stresses from the environment prompt the cells to respond in many ways. The intended outcome of the cell's initial response towards stressors is to defend and recover the cell from the shock, although not all cells will survive [1]. As such, cellular responses are divided into two types. The first is the survival pathways activation, and the second is the commencement of cell death which is intended to remove damaged cells [1]. Cellular responses can also be triggered by the application of physical stimuli, which activates mechanosensory proteins; a phenomenon referred to as “mechanotransduction” [2, 3]. Notably positive responses to low‐frequency mechanical vibrations by healthy cells including bone formation and gene expression by primary and cultured osteoblast in mice model, and even recovery of stem cell proliferation through Linker of Nucleoskeleton and Cytoskeleton (LINC) complex [4, 5]. On the contrary, this stimulus seems to impact cancer cells negatively, including A431 [6]. Our previous study established that mechanical vibration of 20 Hz led A431 cells to death by apoptosis pathway within 48 h after the stimulus [6]. Therefore, this approach is useful as A431 cell line therapy, which may find application in human carcinoma. Furthermore, prior to apoptosis, mechanical vibration changes the glucose consumption pattern of stimulated A431 [7, 8], which signifies A431 cellular metabolic stress. However, the mechanotransduction cascades which underlie the presumed metabolic or other cellular stress in A431 are currently not very well known yet.

As one of the major factors for cellular metabolic stress response upon mechanical stimuli, reactive oxygen species (ROS) play a role in the activation of cell signaling cascades, including triggering cell death at high ROS concentrations [9]. The increase of ROS levels in cancer cells goes hand in hand with the occurrence of mitochondrial apoptosis [10]. In the condition of low ATP and high levels of ROS, cancer cells are set off to enter the death phase [11]. Therefore, cancerous cells increase cellular glucose metabolism or glycolysis at the initial phase of oxidative stress as a response to the dramatic rise of ROS concentrations [12, 13]. Other than metabolic and oxidative stress reactions, measuring stress responses in A431 cells can be done by evaluating the expression of their endogenous genes. Heat Shock Protein 70 (HSP70) is an ATP‐dependent large protein that helps balance protein modulation [14]. The production of HSP70 by the *HSPA1A* gene is very crucial to keeping organisms alive during hyperthermia and hypoxia [15], including A431 cells [16]. High Mobility Group Box 1 (HMGB1) is a Damage‐associated Molecular Pattern (DAMP) protein, which is expressed by the *HMGB1* gene as a signal of dying cells [17]. It was released into the growth media of apoptotic A431 cells, 48 h after low‐frequency mechanical vibration stimulation, in lesser amounts compared to non‐stimulated A431 [6]. Thus, *HSPA1A* and *HMGB1* genes might be upregulated in A431 following extracellular stress.

This study aims to reveal the effect of 20 Hz mechanical stimulation on A431 in the condition of metabolic and oxidative stress. The incubation condition prior to 20 Hz mechanical vibration was made serum‐free as done in a previous study [6] with additional stress, that is, less glucose concentration in 1.0 g·L^−1^, as tumorigenic cells mainly depend on glucose and amino acids as the primary nutrient source [18]. The A431 was subjected to 1 h of mechanical stimulation at 20 Hz after 24 h of incubation. The fate of cancer cell lines, including A431, can be controlled by 20 Hz vibration, mainly to avoid extreme temperature rise and cell necrosis [19], which, in principle, was also applied to cancer cells by using magneto‐mechanical vibration [20]. In terms of oxidative stress signified by ROS expression, a study on MC3T3‐E1 osteoblasts confirmed changes in the ROS regulatory mechanism through vibration stress of 40 Hz with acceleration of 0.49 g and amplitude of ±76 μm for 30 min [21]. Meanwhile, this study utilizes 20 Hz, with an amplitude of ±140 μm and acceleration of 0.23 g for 1 h, which is determined to be comparable to inducing a shift in ROS.

Apart from A431, this study used C2C12 and L929. The two cell lines were chosen due to their properties. The myoblast C2C12 can exhibit immortalized characteristics [22]. Whereas L929, as a fibroblast, can exhibit both immortalized and tumorigenic properties, which make it a model cell line for testing anti‐cancer pharmaceutical therapy [23]. Mechanical stimuli were also proven to induce various signaling cascades for both C2C12 and L929 depending on the types and vibration frequency [24, 25]. Thus, all three cell lines were selected based on their responsiveness to mechanical stimuli. In detail, cell viability, proliferation, glucose consumption, and ROS production were examined in A431, L929, and C2C12 cell lines by taking samples at different time points after vibration. The expressions of HMGB1 and HSP70 coding genes, *HMGB1* and *HSPA1A*, were observed with sampling times of 0 and 24 h after vibration. Lastly, the morphologies of viable A431 cells at 0, 8, and 24 h after mechanical stimulation were observed. The findings of this study may help to clarify the mechanism leading to A431 apoptotic cell death triggered by mechanical vibration.

## Materials and methods

### A431, C2C12, and L929 cell culture

The human epidermoid carcinoma cell line A431 was obtained from RIKEN (Wako, Saitama, Japan). Both the mouse myoblast C2C12 and the mouse fibroblast L929 cell lines were acquired from RIKEN Bio Resource Center (Ibaraki, Japan, with cell codes of RCB0987 and RCB1451, respectively). The A431 cells were cultured in Dulbecco's modified Eagle's medium (DMEM) containing 4.5 g·L^−1^ glucose and 4 mM glutamine (11 995 065; Gibco, Tokyo, Japan), and 10% (v/v) fetal bovine serum (FBS; 11 885 084; Gibco) with 1% penicillin (15 140 122; Thermo Fisher Scientific Inc.). The C2C12 cells were cultured in Dulbecco's modified Eagle's medium (DMEM)/Nutrient Mixture F‐12 Ham with l‐glutamine, 15 mM HEPES (D8900‐1L; Sigma, Tokyo, Japan) supplemented with 10% (v/v) fetal bovine serum. Whereas the L929 cells were cultured in Eagle's minimal essential medium (MEM) “Nissui” (Nissui Pharmaceutical Co., Ltd., Tokyo, Japan) with the addition of 5% (v/v) calf serum. All cell cultures were maintained at 37 °C in a 5% CO_2_ atmosphere. The cells are used after several passages.

### Mechanical stimulation and output vibration

To mechanically stimulate A431, C2C12, and L929 cells, a system of speaker‐based transducer was established. The system was set by connecting a vibration transducer (VLN‐S8BT; Veilnet, Osaka, Japan) via Bluetooth to the controller software (Pd‐extended) installed in a personal computer, as described previously [6]. A 96‐well plate containing the samples was mounted on the transducer. Mechanical vibration in the vertical direction to the culture surface was employed at the bottom of the well plate in sinusoidal waves. The utilized excitation vibration frequency was 20 Hz. The vibration amplitude of each of the 12 wells in the middle section of the 96‐well plate was calibrated using a laser Doppler vibrometer (LV‐1800; Onosokki, Yokohama, Japan) and determined to be 138–140 μm after measurement at the center of each well, as described previously [6]. It was confirmed that pure sinusoidal waves were excited on the culture surfaces.

### Application of mechanical vibration on cell cultures

Before the experiment, cells were seeded into wells at the center of the 96‐well plate at a density of approximately 1 × 10^5^ cells/200 μL in a well for A431 in DMEM with 1.0 g·L^−1^ glucose without FBS. Whereas the cell density for both C2C12 in DMEM/F‐12 Ham and L929 in Eagle's MEM was approximately 1 × 10^4^ cells/200 μL per well. All cells were cultured for 24 h at 37 °C in a 5% CO_2_ atmosphere.

The entire experimental system was then placed in a humidified CO_2_ incubator for 1 h at 34 °C. In the previous study, the medium temperature was confirmed to reach 37 °C during the vibration period due to the heat generated by the vibration transducer [6]. The reason for the 34 °C setting is that when the incubator temperature was set to 37 °C, the medium temperature reached 40 °C within 15 min due to the transducer heat (Table S1). It has been shown that a small change in temperature does not affect cell viability [26]. After the 1 h vibration, test plates were removed from the system and cultured without vibration for a further 8 and 24 h. Control plates were incubated without mechanical vibration for the same time periods.

### Cell counting with trypan blue staining

Cells were harvested from each well of a 96‐well plate by gently pipetting the media in the well up and down several times to ensure all cells were being detached from the well bottom. The cells were mixed thoroughly before transferring 10 μL of the mixture into a 1.5 mL microtube. A volume of 10 μL Gibco Trypan Blue Solution, 0.4% (15 250 061; Thermo Fisher Scientific Inc.) was added into the microtube and mixed with the cells. Subsequently, 10 μL of the suspension was loaded to the counting slide (BioRad, Japan). The total and stained living cells count were obtained by inserting the counting slide to the TC20 Automated Cell Counter (BioRad, Japan).

### Glucose consumption assay

Cell supernatants were collected from the test and control wells at 0, 8, and 24 h after mechanical vibration. In this experiment, the conditions of the environment and the procedures used were the same as the previous work [6]. Glucose concentrations were determined using a Glucose Assay Kit (GAHK‐20; Sigma‐Aldrich, St. Louis, MO, USA) according to the manufacturer's instructions [6].

### Cellular ROS detection assay

ROS produced by each type of cell were measured using the Cellular Reactive Oxygen Species Detection Assay kit (Red Fluorescence) (ab186027; Abcam, UK), based on the manufacturer's instructions. The cells were imaged and analyzed using a high‐content imaging system (BioStation IM‐Q Time‐Lapse Imaging System, Nikon Instruments Inc., Japan) with a CFI Plan Apochromat Lambda D 10x/0.45 objective lens (Nikon, Tokyo, Japan). The ROS expression was determined as the percentage of illuminated area as measured by Image J (National Institutes of Health, New York). The results of ROS expression were normalized to the respective total cell number.

### A431 RNA extraction and cDNA synthesis A431

Relative mRNA expression was measured by RT‐qPCR as described elsewhere [27], using total RNA extracted from the A431 cell line with and without mechanical vibration, at 0 h and 24 h after stimulation. In this study, glyceraldehyde‐3‐phosphate dehydrogenase (GAPDH) was chosen as the housekeeping gene because its expression in A431 was found to be stable under heat, salinity, or other external stressors [28]. The total RNA encoded by *GAPDH*, *HSPA1A*, and *HMGB1* was extracted with a NucleoSpin^®^ RNA (U0955B; Takara Bio, Shiga, Japan) and quantified by RT‐qPCR assay using a Thermal Cycler Dice Real Time System *Lite* (TP700; Takara Bio Inc.). RNA was reverse transcribed into cDNA with PrimeScript RT Master Mix (Perfect Real Time) (RR036A; Takara Bio, Shiga, Japan), using oligo (dT) primer and random hexamer primers. The thermal profile was created with software Takara Thermal Cycler Dice Real Time System Ver. 5.11 for TP700 connected to Thermal Cycler Dice Real Time System *Lite* TP700 (Takara Bio) for 15 min at 37 °C, followed by 5 s at 85 °C, and finally 10 s at 10 °C. The cDNA concentration was quantified using a Biophotometer D30 (6131; Eppendorf, Hamburg, Germany), subsequently diluted to 10 ng·μL^−1^ with RNase‐free water (9012; Takara Bio Inc.).

### RT‐qPCR of A431 genes

The thermal profile for the PCR cycle was configured in TaKaRa Thermal Cycler Dice Real Time System Ver. 5.11 for TP700 software for 30 s at 95 °C, continued with 60 cycles of 5 s at 95 °C and 30 s at 60 °C, subsequently ended by the dissociation curve analysis. The primer sets were supplied by Takara Bio, Shiga, Japan, with the primer sequences shown in Table 1. The final concentration utilized for PCR assays for all primers was 0.3 μM. The RT‐qPCR mix consisted of 12.5 μL TB Green Premix Ex Taq II (Tli RNaseH Plus; RR820A; Takara Bio Inc.), 20 ng cDNA, 0.4 μM of each forward and reverse primer, and 8.5 μL RNase‐free water. The RT‐qPCR was conducted in technical triplicates for each primer pair and cDNA sample. Additionally, the assays were performed as biological triplicates under similar conditions.

**Table 1:** RT‐qPCR primers used in this study.

Gene name	Gene bank number	Sequence (5′‐3′)	Tm (°C)	Product size (bp)
GAPDH	NC_000012.12	Forward GCACCGTCAAGGCTGAGAAC	67.3	138
		Reverse TGGTGAAGACGCCAGTGGA	68.2	
HMGB1	NC_000013.11	Forward AGGATCCCAATGCACCCAAG	68.2	109
		Reverse GCAACATCACCAATGGACAG	68.7	
HSPA1A	NC_000006.12	Forward CCTGGAGTCCTACGCCTTCAAC	68.0	103
		Reverse TTGACACTTGTCCAGCACCTTC	67.1	

Melting curve analysis was carried out for amplicons of each primer pair to ascertain that the fluorescence signals did not come from primer dimers. Negative control reactions without cDNA templates were also incorporated to warrant the assay quality. The results of relative mRNA expressions were normalized to *GAPDH* mRNA and then calibrated to the control, non‐mechanically stimulated A431 cells. The fold change was counted with the 2^−ΔΔCt^ method wherein C_t_ represents the threshold cycle.

### Calcein‐AM staining

The viable A431 cell morphology was analyzed 0, 8, and 24 h after vibration at a cell density of 0.8 × 10^4^ cells/200 μL DMEM with 1 g·L^−1^ glucose without FBS/well. The staining of viable cells may support as visual data of the ratio of viable cell counts. A concentration of 2 μM of ‐Cellstain‐Calcein‐AM (C326, Dojindo, Tokyo, Japan) was added to the media in each well, in dark condition. The plate was incubated for 30 min at 37 °C in a 5% CO_2_ atmosphere. The morphology of the living A431 cells were visualized with an Eclipse Ti‐S inverted microscope (Nikon, Tokyo, Japan) with CFI Plan Apochromat Lambda D 10×/0.45 objective lens. The images were captured using NIS‐Elements D version 4.30 software (Nikon, Tokyo, Japan). Cell viability was determined as the percentage of living cells area percentage, marked by Calcein‐AM stain, within the cell‐occupied proportion measured by imagej.

### Statistical analysis

The data of cell viability ratio, proliferation, glucose consumption, and ROS production are presented as the mean ± standard deviation (SD) of biological triplicates. The differences between group means were evaluated by Welch's *t*‐test, which permits difference in the standard deviations while also possessing comparable power when contrasted to the student's *t*‐test [29]. This test was used to analyze the cell viability ratio, glucose consumption, ROS production, and RT‐qPCR data. *P* < 0.05 was considered statistically significant.

## Results

### A431 cell viability decreases at 24 h after vibration

**Fig. 1:**
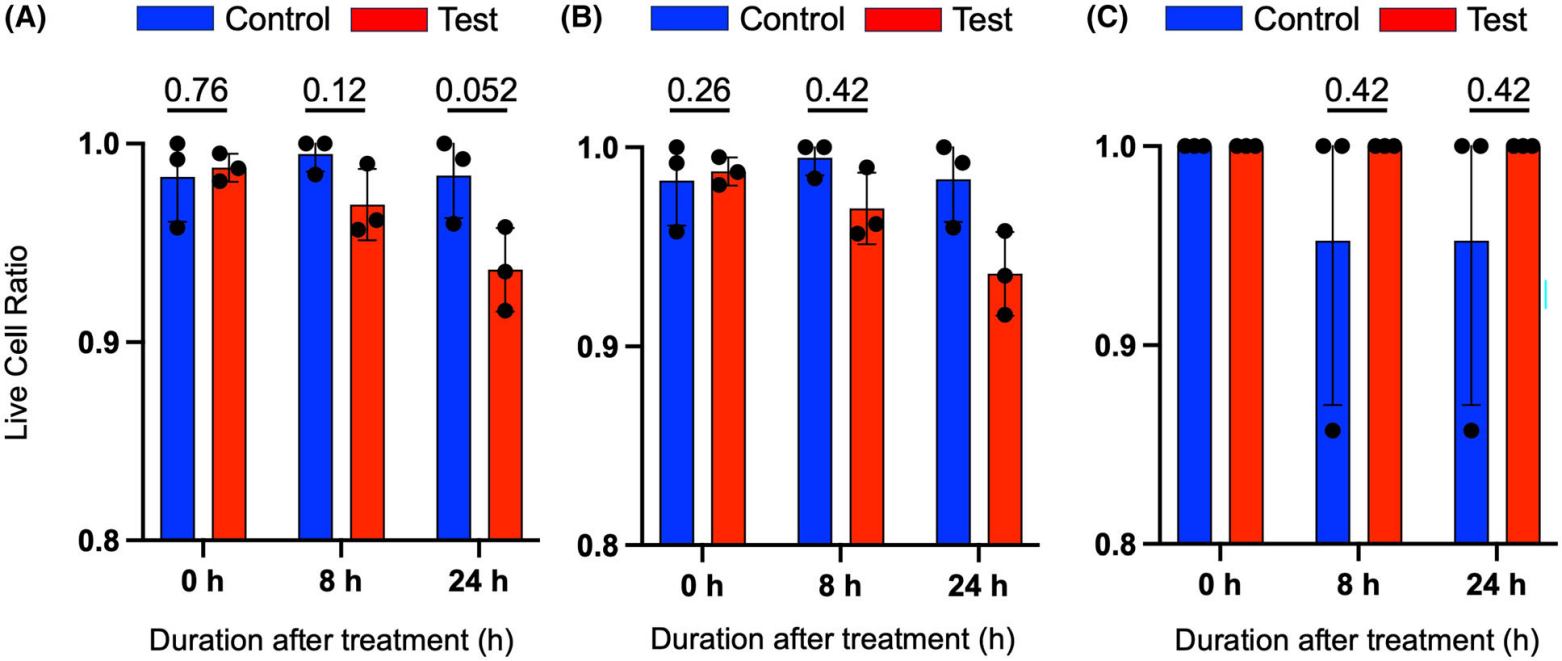
Mechanical stimulation increases the death rate of A431. The numbers supplied above each time point are the *P*‐values. Each *P*‐value was calculated using Welch's *t*‐test. At 24 h, the *P*‐value was 0.052. Cell death and viability ratio was counted for (A) A431, (B) C2C12, and (C) L929 cell lines. Data are presented as the mean ± SD of *n* = 3.

Cell death ratio was examined after mechanical vibration with cell counting using hemocytometer with trypan blue staining. Figure 1A shows that mechanical stimulation promotes more death of A431 cells at 24 h compared to the control, although the difference is not statistically significant. The live cell ratio between the tests and controls of all time points of both C2C12 and L929 did not exhibit noticeable deviation, as shown in Fig. 1B,C. In addition, there is no significant difference between the control and treated C2C12 and L929 cells during the incubation after the treatment period.

### Mechanical vibration slows down glucose uptake by A431

**Fig. 2:**
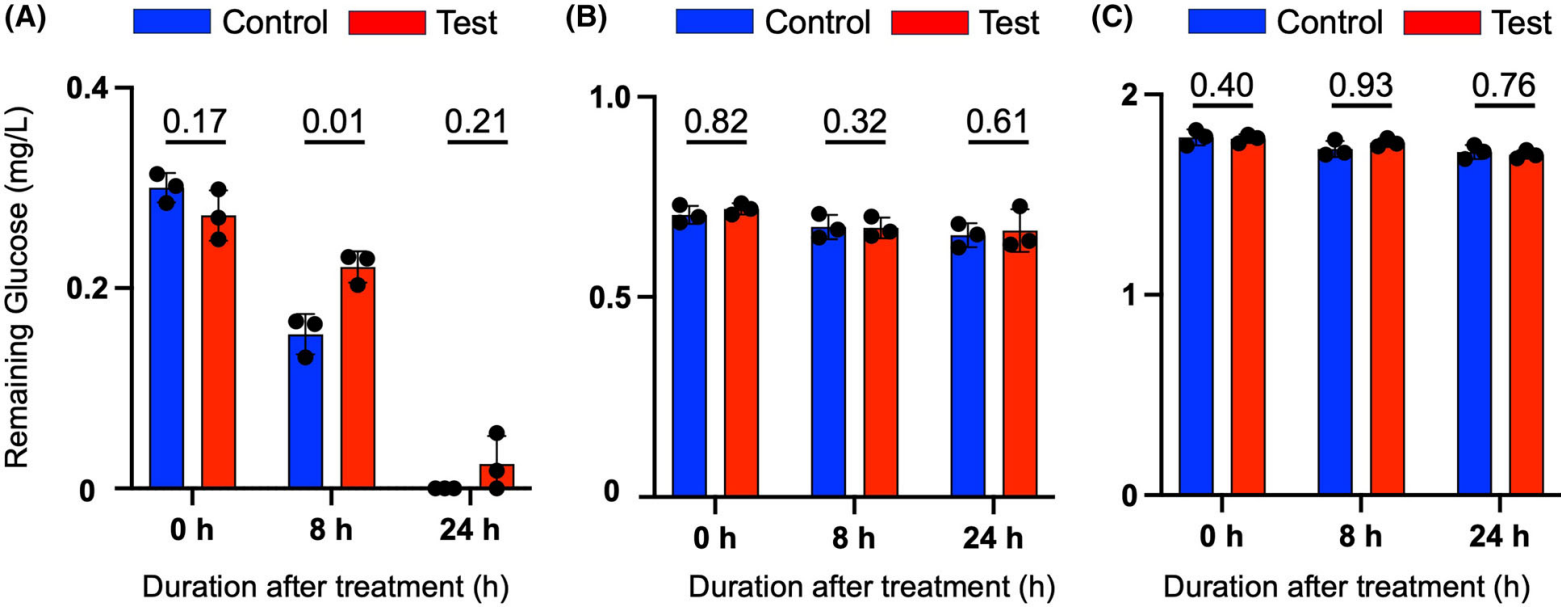
The A431 cells consumed glucose less rapidly after mechanically stimulated, compared to the untreated control and the other cell lines. The numbers above each time point are the corresponding *P*‐values. Each *P*‐value was calculated using Welch's *t*‐test. The *P*‐value at 8 h is statistically significant at 0.01. The amount of remaining glucose in the media was measured for (A) A431, (B) C2C12, and (C) L929. Data are presented as the mean ± SD of *n* = 3.

Figure 2A indicates the glucose level in A431 growth media decreases more rapidly in cells without vibration stimulation. The statistically significant difference is confirmed at the 8 h time point by Welch's *t*‐test. At 24 h after mechanical vibration there was no glucose left in the control sample. In both C2C12 and L929 (Fig. 2B,C), the glucose consumption pattern does not show any observable deviation between the mechanically stimulated sample and the sample without stimulation at all time points. Therefore, it can be said that mechanical vibration contributes specifically to the decrease of glucose ingestion by A431 cells.

### ROS production in A431 decreased upon mechanical stimulation

**Fig. 3:**
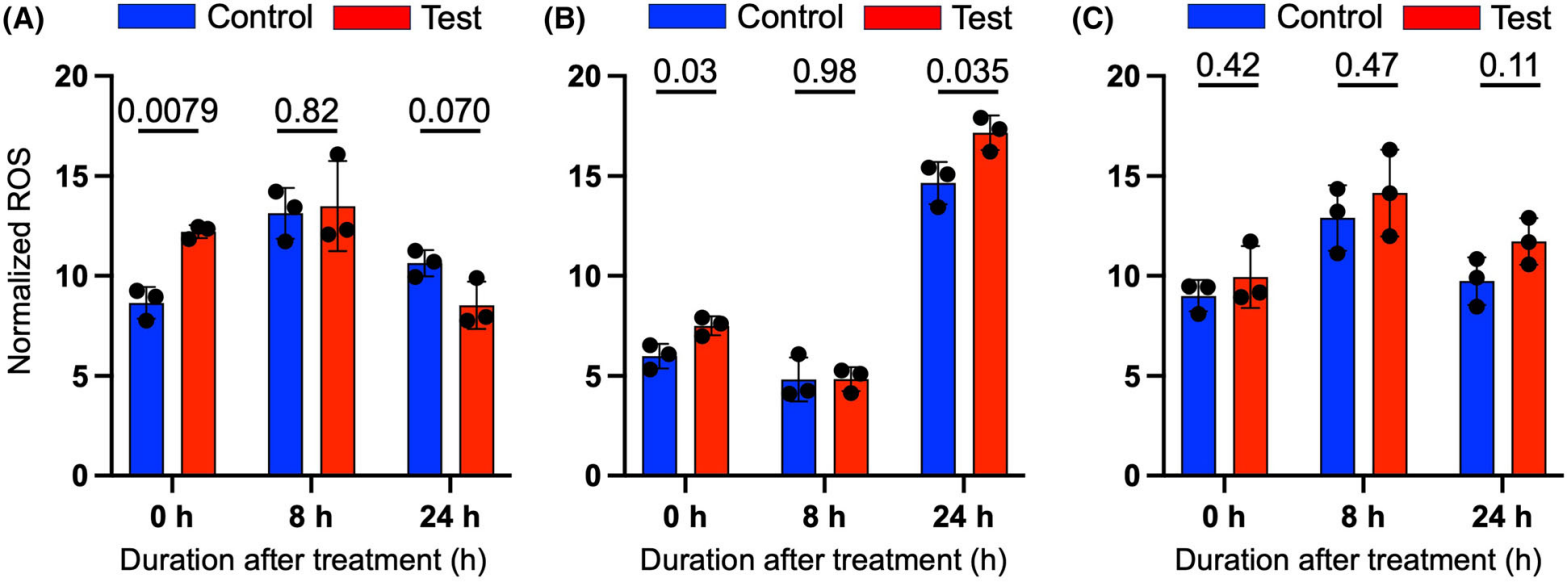
Mechanical vibration stimulates a decrease of ROS production by A431. The numbers given above each time point are the *P*‐values. Each *P*‐value was calculated using Welch's *t*‐test. At 0 h, the *P*‐value of 0.0079 is statistically significant. ROS production was quantified and normalized for A431 as ROS/live cell numbers and data are presented as the mean ± SD of *n* = 3.

The detected ROS expressions were normalized with the corresponding living cells number. The ROS of the mechanically stimulated A431 at 0 h was higher than the control and was confirmed to have a significant difference with the control by Welch's *t*‐test (Fig. 3A). Subsequently, at 24 h, the production of ROS in the test sample decreased and appeared to be lower than the ROS expression of the control as shown in Fig. 3A, although there is no statistically significant difference.

The expression of ROS was also measured for C2C12 and L929 cells, with the results in Fig. 3B for C2C12 and Fig. 3C for L929. For mechanically stimulated C2C12, statistically significant differences were confirmed at 0 h and 24 h after stimulation. At these points, ROS expressions in treated samples are higher than the non‐stimulated ones (Fig. 3B). However, there were no significant differences between the normalized ROS expression of the untreated and treated L929 samples at all time points, as demonstrated in Fig. 3C.

### *RT‐qPCR of* HMGB1 *and* HSPA1A *gene expression in A431*

**Fig. 4:**
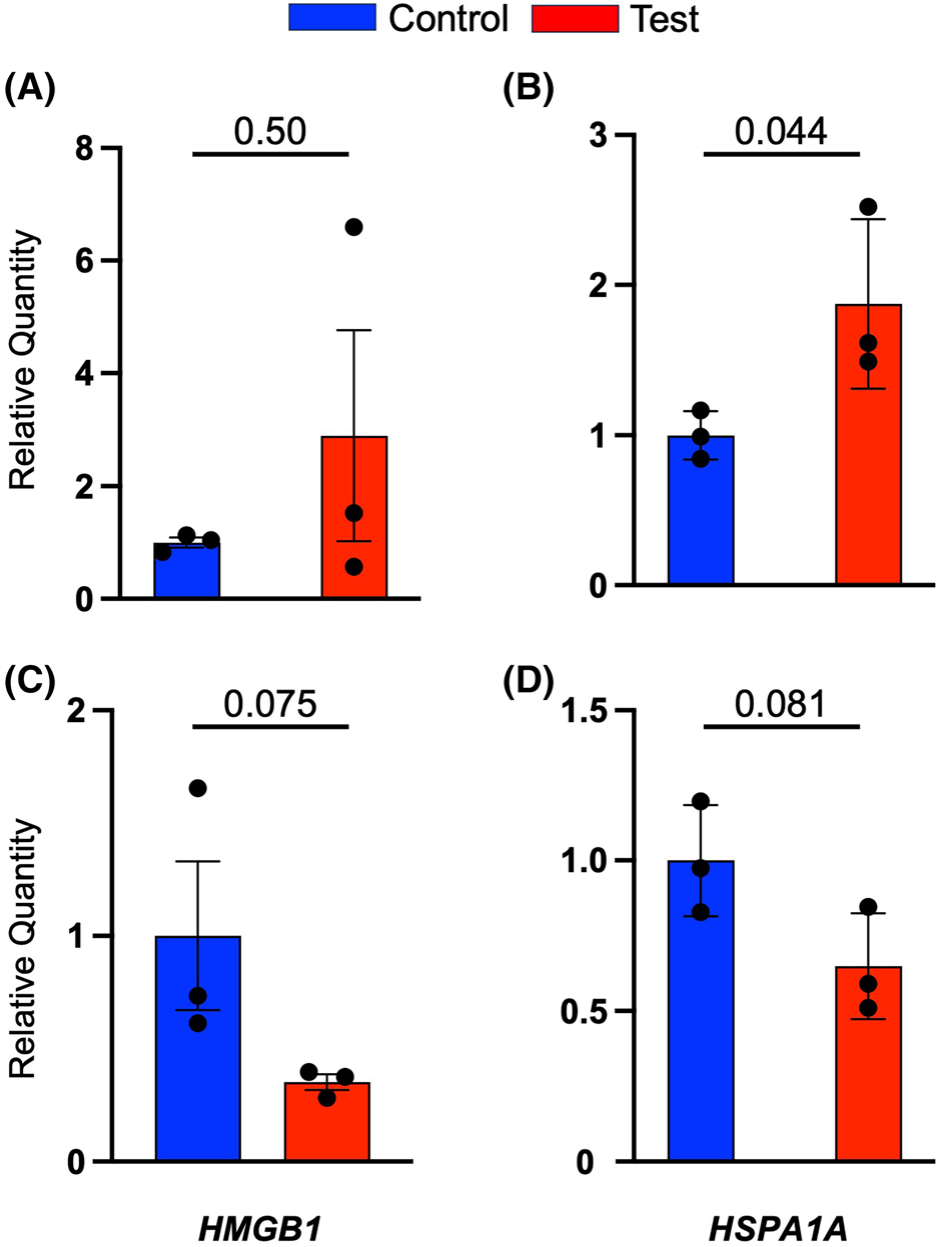
Gene expressions were measured as relative quantities at 0 h after vibration for (A) HMGB1 and (B) HSPA1A genes, at 24 h after vibration for (C) HMGB1 and (D) HSPA1A. The numbers given above each time point and gene type are the *P*‐values. Each *P*‐value was calculated using Welch's *t*‐test. Data are presented as the mean ± SE of *n* = 3.

The quantified results of A431 mRNA expression using RT‐qPCR revealed that right after mechanical vibration of A431 cells (0 h), the expression of the *HMGB1* gene was upregulated relative to the non‐treated cells (Fig. 4A). A similar trend can also be observed in the increase of *HSPA1A* expression (Fig. 4B) although the differences for both cases are not statistically significant. In contrast, 24 h after mechanical stimulation, the expressions of both the *HMGB1* gene and *HSPA1A* gene were downregulated, as can be seen in Fig. 4C,D. Significant differences also could not be statistically confirmed for these samples.

### A431 experiences morphological changes due to mechanical stimulation

**Fig. 5:**
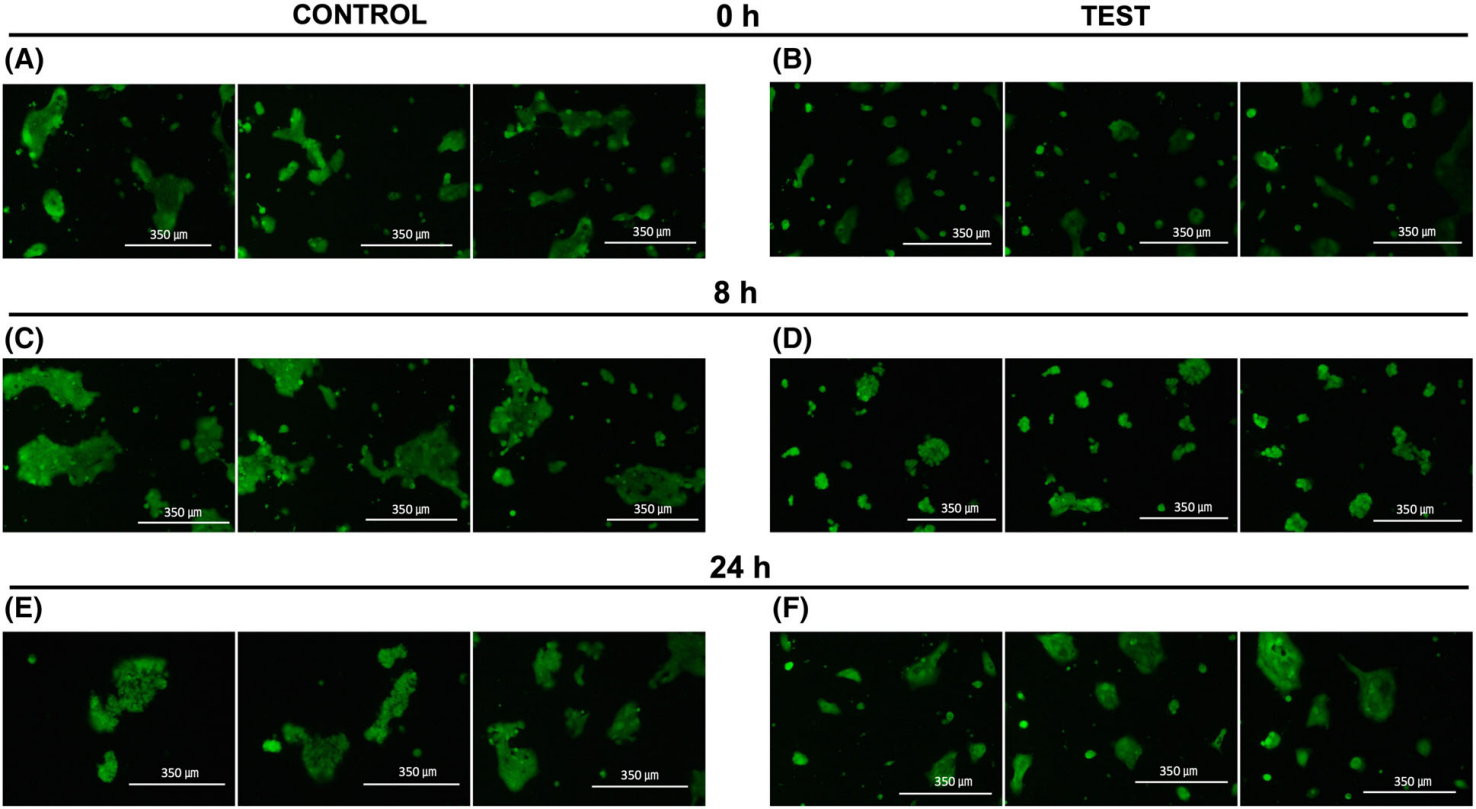
Calcein‐AM staining of A431 at 0 h after vibration of (A) Control and (B) Test, 8 h after vibration of (C) Control and (D) Test, and 24 h after vibration of (E) Control and (F) Test.

The A431 cells were stained with Calcein‐AM to determine the effect of mechanical vibrations on the shape of the cells. After vibration (0 h time point), A431 cells tend to assume rounder shapes (Fig. 5B), in contrast to the non‐stimulated ones which generally form island‐like, elongated colonies in Fig. 5A. It also appears that mechanically stimulated cells exist in smaller and rounder colonies compared to the control sample.

At 8 h time point after vibration, A431 cells joined together and assumed a rounder colony shape, presumably due to the spherical morphology of the individual cells (Fig. 5D). Although the size of the colony increases, the overall shape of the colony is still rounder compared to control cells in Fig. 5C. The condition at the 8 h time point does not seem to differ much from that of the 24 h after vibration. A few A431 cells still appear to stay in individual form or in smaller colonies (Fig. 5F). Meanwhile, the size of non‐stimulated A431 colonies seems to be increasing and there are fewer smaller colonies (Fig. 5E) compared to the control sample of previous time points visualized in Fig. 5A,C.

## Discussion

In this study, we found that low‐frequency mechanical vibration of 20 Hz triggers metabolic and oxidative stresses of A431, prior to cell death. Our results indicated that the mechanical vibration treatment promoted A431 death within 24 h after vibration in comparison to non‐treated cells (Fig. 1A), while the healthy cells, C2C12 and L929, responded differently to the treatment. After incubating the cells prior to mechanical vibration, the case of cell death was not apparent in both C2C12 (Fig. 1B) and L929 (Fig. 1C) within 24 h. As reported previously, C2C12 and L929 were responsive to different mechanical stimuli, such as 0.5 Hz cyclic mechanical stress and 0.2–0.6 W·cm^−2^ ultrasound, respectively [24, 25]. Hence, these cells have the potential to show the effect of mechanotransduction. Additionally, as higher expression of ROS in cancer cells happens simultaneously with the cell's death phase [11], the spike of ROS expression at 0 h confirmed by Welch's *t*‐test (Fig. 3A) may indeed promote A431 cell death within 24 h after mechanical stimulation (Fig. 1A). It should be noted that in this work, the vertical mechanical vibration was employed on the culture surface. On the other hand, there was a study which utilized horizontal vibration [30], and hence this way can be considered in future work.

Tumorigenic aberrant glycolysis produces a lower amount of ATP per glucose molecule compared to normal cells. Thus, the rising uptake of glucose commonly happens in cancer [7]. Interestingly, however, Fig. 2A demonstrates that mechanical vibration slows down glucose consumption by A431 in comparison to the control sample, particularly at 8 h, which is confirmed by Welch's *t*‐test to be significantly different. This result is different from our previous study, published in 2020 [6], where a decreasing rate of glucose intake happened at 24–48 h after vibration. The most probable cause of such difference is the available glucose in incubation media prior to mechanical treatment. The 2020 study used 4.5 g·L^−1^ glucose with 48 h of incubation, while this study utilized 1.0 g·L^−1^ glucose with 24 h incubation. Incubation media of both studies are serum‐free, which limits the availability of essential amino acids for cell nutrients. The presence of amino acids is crucial for cancer cell survival [31]. What possibly happens is that less glucose concentration, coupled with lack of essential amino acids and combined with 20 Hz mechanical vibration, fastens the A431 metabolic and oxidative stress, which prompts the slowing down of glucose consumption rate right after the stimulation (Fig. 2A).

Furthermore, a review by Zanotelli *et al*. suggested increasing evidence on how mechanical signals can stimulate glucose‐related metabolic changes to regulate cancer progression and migration. However, the detailed metabolic process or the impacted pathways remain largely unknown [32]. One of the possible reasons for the decrease in glucose uptake is the low level of Glucose Transporter 1 (GLUT1) [8], whose expression is affected by mechanical stress. Upregulation of GLUT prevents apoptosis [18]; therefore, inversely, its decrease may trigger apoptotic cell death. Further research is necessary to confirm the expressions of such proteins and their effects on A431 cell viability.

RT‐qPCR results of *HMGB1* and *HSPA1A* demonstrated that both genes were upregulated right after mechanical vibration stimulation, as shown in Fig. 4A,B. The increase of both genes' expression at 0 h signified that A431 is overall under a stressful condition triggered by mechanical stimulation. Indeed, the synthesis of both HMGB1 and HSP70 proteins, which are encoded by *HMGB1* and *HSPA1A* genes, respectively, is closely linked to cellular stress [15, 17]. Besides its commonly known role as a cellular damage‐associated protein, a previous study indicated that HMGB1 expression is specifically responsive to mechanical stress [33]; therefore, it is valid to see the increase of *HMGB1* relative quantity directly after vibration (Fig. 4A). The high expression of the *HSPA1A* gene, as confirmed by RT‐qPCR, may be responding to the need for cell survival aided by HSP70 protein [15]. Wong *et al*. determined that the increasing synthesis of HSP70 due to mechanical stress contributes to tumor initiation and progression [34]. Similar to the *HMGB1* gene, the *HSPA1A* gene may also be overexpressed as a direct response to the 20 Hz mechanical vibration (Fig. 4A,B). However, both gene expressions at 24 h after vibration were less than control A431 (Fig. 4C,D). The spike of ROS production at 0 h due to the mechanical stimulation, as pointed out in Fig. 3A, may correlate with the high expression of *HMGB1* and *HSPA1A* at the same time point. Both results confirmed that mechanical stimulation caused the A431 cellular stress‐related signaling cascade.

The effect of mechanical vibration towards the morphology of viable A431 was confirmed by cell staining with Calcein‐AM, which specifically stains living cells [35]. As suggested by the results in Fig. 5, mechanical vibration seems to separate the living A431 into smaller colonies, in contrast to the non‐treated cells. It is known that cells respond to extracellular stimulation, which alters their surface tension and changes their shape [36]. Specifically, mechanical vibration was found to increase cell surface tension, which is likely to change the shape and function of the affected cells [37, 38]. Although the effect of low‐frequency mechanical vibration towards the A431 cytoskeleton network has not been discovered yet, current studies highlighted the effect of such mechanical stimulus towards the cell's cytoskeleton. Research in human keratinocytes (HaCat) found that low intensity vibration rearranges HaCat's cytoskeleton in connection to its nucleus regulated by the Linker of Nucleoskeleton and Cytoskeleton (LINC) complex [39]. Similarly, it is thought that A431 may also undergo shifts in its cytoskeleton network; hence, the morphological changes, due to the 20 Hz mechanical vibration. Cell death by apoptosis happens concurrently with cell shrinking and rounding, followed by cytoskeleton detachment from the membrane which will lead to the membrane blebbing phenomenon [40]. A431 experienced apoptosis by means of mechanical stimulation at 20 Hz during 24–48 h after the treatment [6]. The observed rounding and shrinking phenomenon as described in Fig. 5C, especially at the 24 h time point, may well be related to the onset of apoptosis. Similarly, the increasing cell death at 24 h after mechanical stimulation (Fig. 1A) and the decrease of gene expressions at 24 h (Fig. 4C) compared to the expressions at 0 h are likely to be caused by apoptosis of A431.

## Conclusion

Mechanical vibration at 20 Hz promotes metabolic and oxidative stresses in A431 by slowing down glucose intake and increasing ROS production. The two phenomena, along with the overexpression of HMGB1 and HSP70 coding genes 0 h after stimulation, might lead to A431 cell death, presumably by the apoptotic pathway. The suggestion that several pathways are involved and converging to trigger the cancer cell death, supported by the results of this study, is in line with the hypothesis in our previous work [6]. We also have examined the effect of mechanical vibration on C2C12 and L929, as recommended in our previous study. None of the normal cells display any remarkable change in behavior upon receiving 20 Hz mechanical stimulations. Thus, it can be concluded that 20 Hz mechanical vibration exclusively triggers cellular stress which initiates carcinoma death by apoptosis, in this case A431, strengthening the potential of this biomechanical treatment in cancer therapy.

## Conflict of interest

The authors have declared no conflicts of interest.

## Peer review

The peer review history for this article is available at https://www.webofscience.com/api/gateway/wos/peer‐review/10.1002/2211‐5463.70055.

## Author contributions

WLA, CI, and TM designed and performed all experiments. SM and AF provided concepts and supervised the experiments. KT supervised overall research and devised the concept for the initial research design. All authors contributed to the analysis and interpretation of the data, as well as the preparation of the manuscript.

## Supporting information


**Table S1.** Pilot experiment to determine incubator temperature for a constant final media temperature of 37.0 °C. The plate used was a pre‐incubated, 37.0 °C plate with media of the same temperature. The cell marked with asterisk (*) represented a trial being stopped due to temperature of almost 40 °C within 15 min. In that condition, the cell layer peeled from the well bottom. The media temperature was monitored with measuring device TR‐71wb (T&D Holdings, Inc., Tokyo, Japan).

## Data Availability

The data that support the findings of this study are available from the corresponding author upon reasonable request.
