# Supplementary material for: Profiling the effect of low frequency mechanical vibration on the metabolic and oxidative stress responses of A431 carcinoma
Source: FEBS Open Bio. 2025 May 16;15(8):1365–75. doi: 10.1002/2211-5463.70055 (PMC12319701; doi:10.1002/2211-5463.70055)
Supplement: Supplementary file 1 — Table S1. Pilot experiment to determine incubator temperature for a constant final media temperature of 37.0 °C. The plate used was a pre‐incubated, 37.0 °C plate with media of the same temperature. The cell marked with asterisk (*) represented a trial being stopped due to temperature of almost 40 °C within 15 min. In that condition, the cell layer peeled from the well bottom. The media temperature was monitored with measuring device TR‐71wb (T&D Holdings, Inc., Tokyo, Japan). [file FEB4-15-1365-s001.docx]

**Supplementary Materials**

**Table S1.** Pilot experiment to determine incubator temperature for a constant final media temperature of 37.0^o^C. The plate used was a pre-incubated, 37.0^o^C plate with media of the same temperature. The cell marked with asterisk (*) represented a trial being stopped due to temperature of almost 40^o^C within 15 mins. In that condition, the cell layer peeled from the well bottom. The media temperature was monitored with measuring device TR-71wb (T&D Holdings, Inc., Tokyo, Japan).

| Humidified incubator temperature | Plate bottom and media temperature | | | | |
| --- | --- | --- | --- | --- | --- |
|  | 0 min | 15 mins | 30 mins | 45 mins | 60 mins |
| 37.0^o^C | 37.0^o^C | (*)39.7^o^C | - | - | - |
| 35.0^o^C | 37.0^o^C | 37.2^o^C | 37.6^o^C | 37.9^o^C | 37.9^o^C |
| 34.0^o^C | 37.0^o^C | 36.9^o^C | 36.9^o^C | 37.0^o^C | 37.0^o^C |
